# Evaluation of serum epidermal growth factor receptor (EGFR) in correlation to circulating tumor cells in patients with metastatic breast cancer

**DOI:** 10.1038/s41598-017-17514-8

**Published:** 2017-12-11

**Authors:** Malgorzata Banys-Paluchowski, Isabell Witzel, Sabine Riethdorf, Brigitte Rack, Wolfgang Janni, Peter A. Fasching, Erich-Franz Solomayer, Bahriye Aktas, Sabine Kasimir-Bauer, Klaus Pantel, Tanja Fehm, Volkmar Müller

**Affiliations:** 1 Department of Gynecology and Obstetrics, Marienkrankenhaus Hamburg, Hamburg, Germany; 2 Department of Gynecology, University Medical Center Hamburg-Eppendorf, Hamburg, Germany; 3 Department of Tumour Biology, University Medical Center Hamburg-Eppendorf, Hamburg, Germany; 4 Department of Gynecology and Obstetrics, University Hospital Ulm, Ulm, Germany; 5 Department of Gynecology and Obstetrics, University Erlangen, Erlangen, Germany; 6 Department of Gynecology and Obstetrics, Saarland University Hospital, Homburg/Saar, Germany; 7 Department of Obstetrics and Gynecology, University Hospital Leipzig, Leipzig, Germany; 8 Department of Obstetrics and Gynecology, University Hospital Essen, University of Duisburg-Essen, Essen, Germany; 9 Department of Obstetrics and Gynecology, Heinrich-Heine-University Düsseldorf, Düsseldorf, Germany

## Abstract

Overexpression of epidermal growth factor receptor in breast cancer is associated with estrogen receptor negativity, higher histological grade and larger tumors. The aim of the present study was to evaluate the clinical significance of serum EGFR (sEGFR) in relation to circulating tumor cells (CTCs) in metastatic breast cancer. 252 patients were enrolled in this prospective multicentre study. Blood was drawn before start of a new line of therapy. sEGFR was determined using a sandwich-type ELISA. CTCs were detected using CellSearch. sEGFR was determined in 48 healthy controls and 252 patients, with no significant differences between the two groups. Clinical-pathological parameters did not correlate with sEGFR, irrespective of the cutoff chosen. Patients with sEGFR levels above the 50^th^ and 75^th^ percentile were more likely to present with <5 CTCs per 7.5 ml blood (p = 0.007; p = 0.003). Patients with sEGFR ≥73 ng/ml had significantly longer overall survival than those with sEGFR <73 ng/ml (19.7 vs. 15.2 months; p = 0.007). In the multivariate analysis, presence of ≥5 CTCs, higher grading and higher line of therapy remained independent predictors of shorter OS, while only higher line of therapy and presence of ≥5 CTCs were independent predictors of shorter PFS.

## Introduction

The family of erbB receptors consists of four closely related transmembrane proteins (erbB1, erbB2, erbB3, erbB4) involved in a network of signalling pathways that have been shown to play a major role in malignant transformation of various epithelial tumors^1–4^. In breast cancer (BC), the most extensively studied member of the erbB family is the erbB2 receptor, also known as HER2, which is overexpressed by 15–20% of primary tumors and serves as a target for highly effective antibody-based therapies. While evidence exists to support HER2 activity to be directly linked to enhanced mobility and invasiveness of cancer cells, data on the clinical relevance of the first discovered protein of the erbB family, the erbB1 receptor, is less conclusive^5^. Commonly referred to as the epidermal growth factor receptor (EGFR), erbB1 undergoes conformational changes upon binding of a specific ligand, inducing downstream signal transduction by various pathways.

EGFR expression in the tumor tissue can be assessed by immunohistochemistry and *in situ* hybridization, resulting in overexpression rates in BC patients ranging from 6 to 60%, depending on the method used^6–11^. EGFR overexpression has been shown to correlate with higher histological grade and estrogen receptor (ER) negativity; larger and inflammatory tumors are more likely to be EGFR-positive^7,11,12^. The majority of published studies reported worse clinical outcome in patients with EGFR-overexpressing tumors^13–15^. Since all erbB receptors share a similar structure containing an extracellular ligand-binding domain (ECD) that may be shed from the cell surface into the blood stream, attempts have been made to measure EGFR levels in the serum using enzyme-based assays and evaluate its clinical utility as a biomarker^16–18^. Interestingly, studies have shown that BC patients have lower serum EGFR (sEGFR) levels than age-matched healthy controls, while in other tumor entities, such as glioblastoma and head and neck squamous cell carcinoma, sEGFR levels in patient samples were significantly higher than in controls^17,19–22^. Decreased sEGFR levels predicted shorter overall survival in metastatic BC in several trials, however, survival did not correlate with sEGFR in other studies^17,18,23,24^. Therefore, the prognostic relevance of sEGFR remains to be further clarified.

In the context of blood-based biomarkers, detection of circulating tumor cells (CTCs) is currently the most promising tool to predict prognosis and monitor treatment with a large body of evidence in both early and metastatic BC^25–27^. While small studies have aimed at exploring the relationship between sEGFR and CTCs in lung and colorectal cancer, data on breast cancer are limited. The aim of the present study was to evaluate the clinical significance of sEGFR levels in relation to the CTCs in a large cohort of metastatic BC patients.

## Results

### Patients’ characteristics

**Figure 1:**
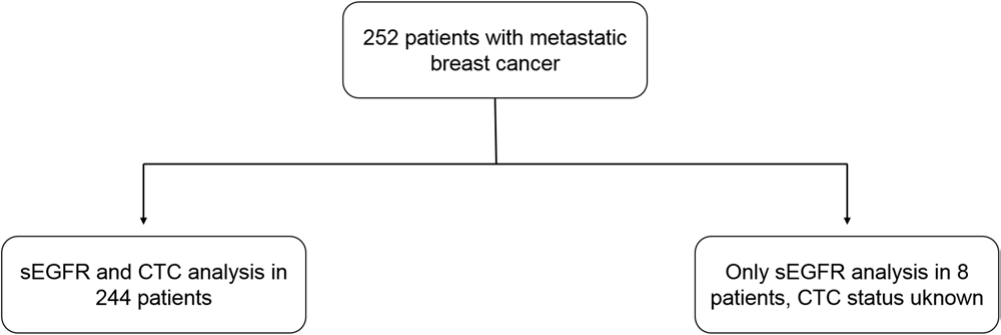
Patient distribution diagram according to the REMARK criteria.

252 patients diagnosed with metastatic breast cancer were included into the analysis. Clinical-pathological data are summarized in Table 1. The median age of patients was 60 years. HER2 was overexpressed by the primary tumor and/or metastasis in 35% of patients; the majority of patients (70%) had a hormone receptor positive tumor. 49.8% of patients presented with ≥ five CTCs per 7.5 ml of peripheral blood. The distribution of patients is summarized in a REMARK diagram (Fig. 1).

**Table 1:** Patients’ characteristics (significant values are shown in bold).

	Total	EGFR ≥73 ng/ml n (%)	p-value
**Overall**	252	63 (25%)	
ER status			0.542
Negative	76	21 (28%)	
Positive	175	42 (24%)	
**PR status**			0.192
Negative	102	30 (29%)	
Positive	149	33 (22%)	
**HER2 status**			0.722
Negative^1^	143	37 (26%)	
Positive^2^	76	18 (24%)	
**Metastatic site**			0.951
Visceral	99	25 (25%)	
Bone	35	8 (23%)	
Both	118	30 (25%)	
**Extent of metastatic disease**			0.489
One site	85	19 (22%)	
Multiple sites	167	44 (26%)	
**Therapeutic setting**			0.206
1st-line	98	29 (30%)	
2nd-line	66	17 (26%)	
3rd-line or more	87	16 (18%)	
**Grading**			0.851
G1	6	2 (33%)	
G2	129	34 (26%)	
G3	103	25 (24%)	
**Circulating tumor cells**	<5		**0.003**
<5 CTCs/7.5 ml	122	41 (34%)	
<5 CTCs/7.5 ml	122	21 (17%)	
**Serum HER2**			0.245
≥15 ng/ml	119	26 (22%)	
≥15 ng/ml	131	37 (28%)	

^1^IHC score: 0/+ 1 or FISH negative,^2^ICH score: + 3 or FISH positive.

### sEGFR detection in BC patients and healthy controls

Levels of sEGFR were determined in 48 healthy controls and 252 patients, with no significant differences between the two groups (independent samples t-test; Table 2). Initially, six cutoffs were considered; two were based on the analysis of healthy controls (mean +2 × standard deviation: 97 ng/ml and mean −2 × standard deviation: 29 ng/ml), three on the analysis of the patient cohort (25^th^ percentile: 54 ng/ml, 50^th^ percentile: 62 ng/ml, 75^th^ percentile: 73 ng/ml). In addition, the previously reported cutoff of 45 ng/ml was considered as well^17,21^. All patients had sEGFR levels above 29 ng/ml, only 9% patients had sEGFR levels above 97 ng/ml and only 9% lower than 45 ng/ml (Table 3). Clinical-pathological parameters, such as hormone receptor and HER2 status, line of therapy, extent of disease and grading, did not correlate with sEGFR levels, irrespective of the cutoff chosen. No correlation was found between serum HER2 levels and sEGFR. Patients with sEGFR levels above the 50^th^ and 75^th^ percentile were more likely to present with <5 CTCs per 7.5 ml blood (p = 0.007 and p = 0.003, respectively; chi-square test). Table 1 shows the results for the cutoff of 73 ng/ml, i.e. 75^th^ percentile.

**Table 2:** Evaluation of sEGFR in blood samples of healthy controls and metastatic breast cancer patients.

	Healthy controls	Patients
Number of samples	48	252
Mean	62.74 ng/ml	66.88 ng/ml
Median	60.35 ng/ml	62.00 ng/ml
Range	38.11–126.70 ng/ml	30.00–176.90 ng/ml
Standard deviation	17.06 g/ml	21.26 ng/ml

**Table 3:** Comparison of different cutoff values for sEGFR.

Cutoff value (ng/ml)	28.6	45	54	62	73	96.8
Basis for the cutoff	Mean −2 × standard deviation in healthy controls	Used by other authors for the same assay system^17,21^	25^th^ percentile in patient cohort	50^th^ percentile in patient cohort	75^th^ percentile in patient cohort	Mean +2 × standard deviation in healthy controls
Healthy controls: samples above the cutoff	100%	94%	71%	35%	17%	4%
Metastatic BC patients: samples above the cutoff	100%	91%	75%	50%	25%	9%
PFS (univariate analysis)	—	P = 0.088 (shorter in low sEGFR)	P = 0.740	P = 0.862	P = 0.270	P = 0.634
OS (univariate analysis)	—	P = 0.384	P = 0.446	P = 0.057 (shorter in low sEGFR)	P = 0.007 (shorter in low sEGFR)	P = 0.368
OS (multivariate analysis)	—			n.s.	n.s.	
Correlation with CTCs	—	P = 1.0	P = 0.372	P = 0.007 (elevated sEGFR in 58% of CTC-neg patients vs. 41% of CTC-pos)	P = 0.003 (elevated sEGFR in 34% of CTC-neg patients vs. 17% of CTC-pos)	P = 0.049 (elevated sEGFR in 6% of CTC-neg patients vs. 13% of CTC-pos)

### Univariate survival analysis

**Figure 2:**
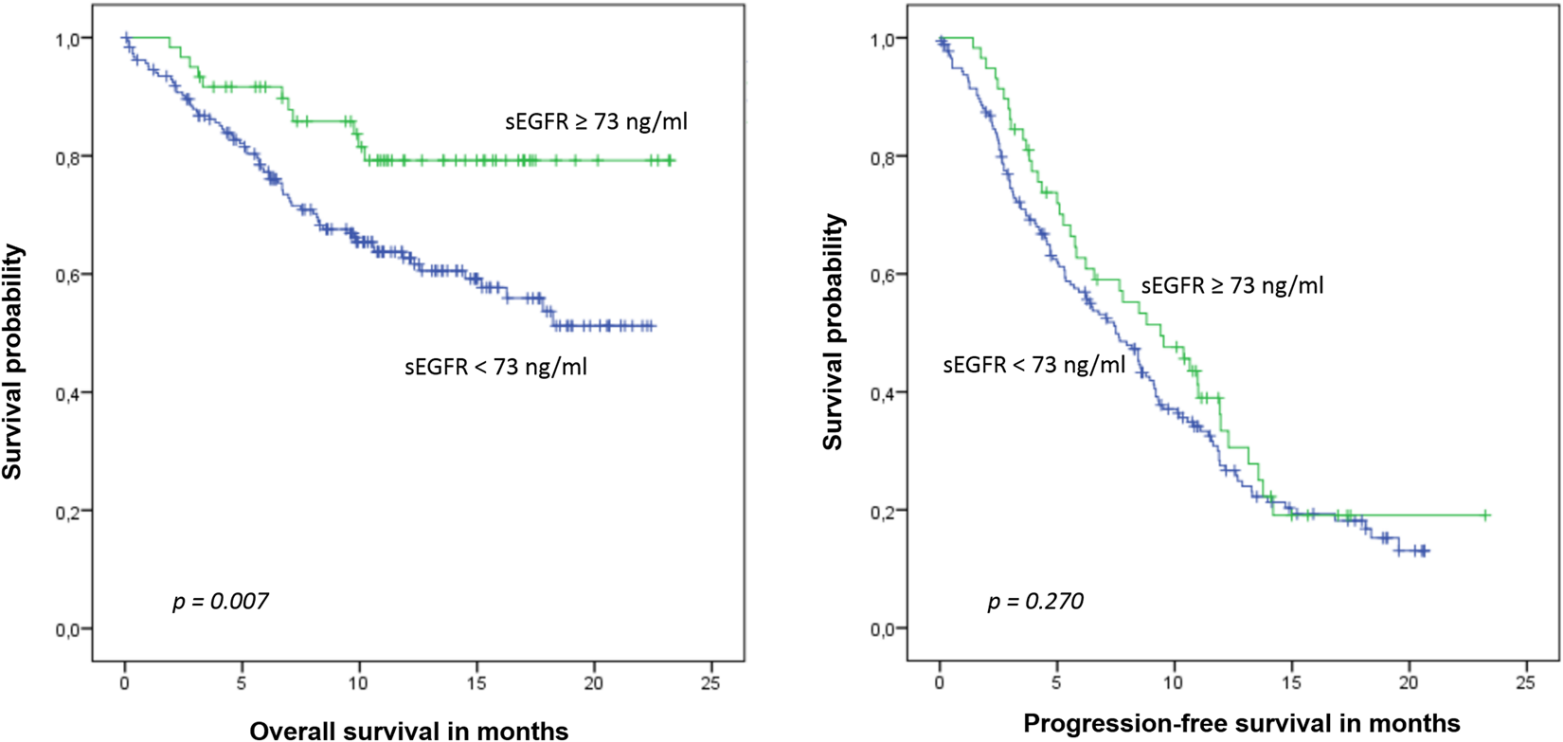
Correlation between overall and progression-free survival and sEGFR levels using the cutoff of 73 ng/ml (75^th^ percentile).

**Figure 3:**
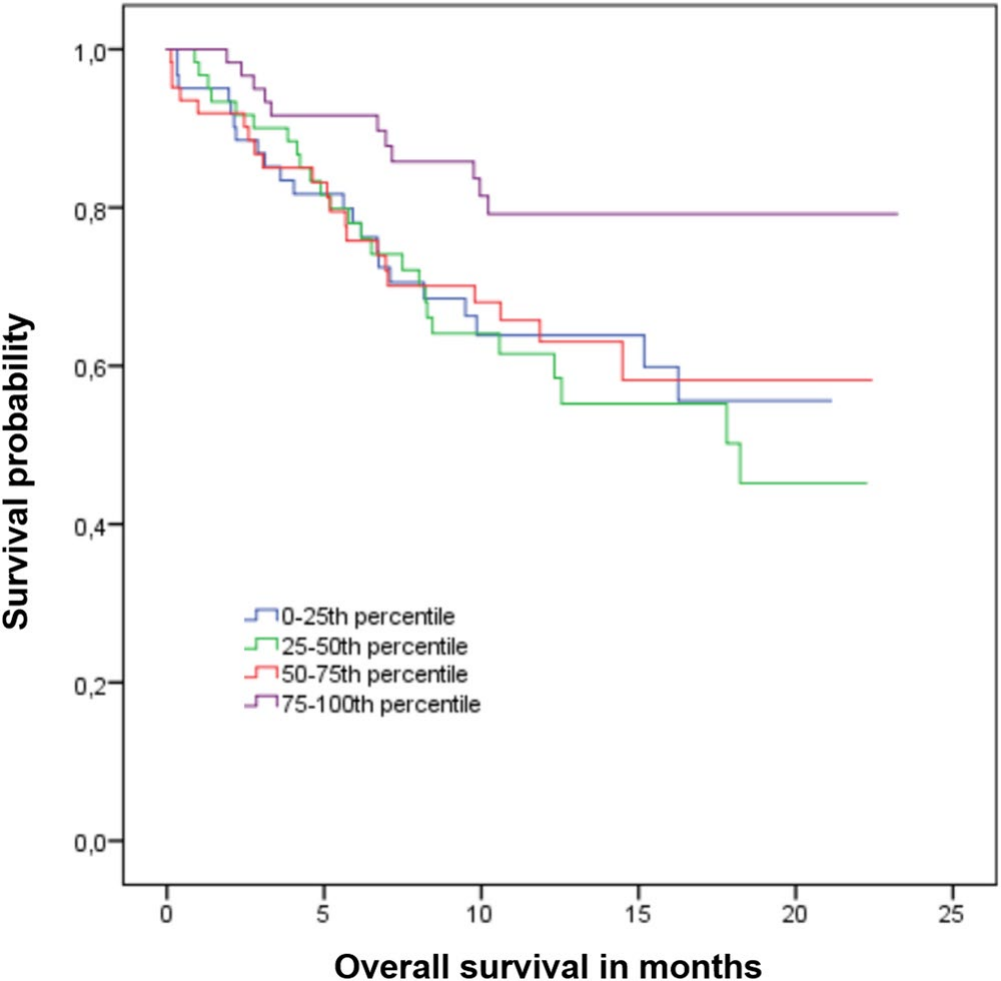
Kaplan-Meier plot of overall survival stratified by quartiles of sEGFR levels.

During a median follow up of 19 months, 85 patients died and 183 were diagnosed with progressive disease. Mean overall survival (OS) was 19.7 months (95%-CI: 17.8–21.6 months) in patients with sEGFR ≥73 ng/ml versus 15.2 months (95%-CI: 13.9–16.5) in patients with sEGFR levels <73 ng/ml; median OS has not been reached in either group (p = 0.007) (Fig. 2). No significant impact on OS was observed when other cutoffs were considered (p = 0.057 for 62 ng/ml and p = 0.446 for 54 ng/ml, respectively; Fig. 3). Median progression-free survival (PFS) was 9.4 months (95%-CI: 6.1–12.6) for patients with sEGFR ≥3 ng/ml versus 7.5 months (5.9–9.0 months) with sEGFR levels <73 ng/ml (p = 0.270) (Fig. 2). No significant correlation between PFS and sEGFR was found when other cutoffs were used (Table 3).

**Figure 4:**
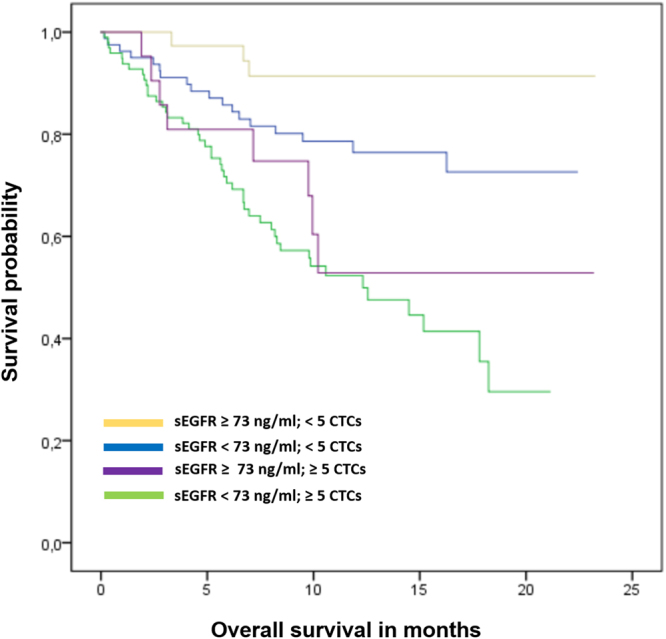
Kaplan-Meier plot of overall survival according to the combined analysis of circulating tumor cells and sEGFR levels.

As reported previously, positive CTC status was significantly associated with shorter PFS (p = 0.001) and OS (p < 0.001). Patients with sEGFR <73 ng/ml and ≥5 CTCs had the shortest OS (mean 12.3 [95%-CI: 10.6–14.0] months, median 12.3 [95%-CI: 7.5–17.2] months), while patients with sEGFR ≥73 ng/ml and <5 CTCs had the longest OS (mean 21.7 [20.1–23.4] months, median not reached; p < 0.001; Fig. 4).

### Multivariate survival analysis

Classical clinical-pathological factors as well as CTC status, sEGFR and sHER2 were included into a multivariate Cox regression analysis. In the multivariate analysis, presence of ≥5 CTCs, higher grading and higher line of therapy remained independent predictors of shorter OS. Only higher line of therapy and elevated CTC counts were independent predictors of shorter PFS in the multivariate analysis (Table 4).

**Table 4:** Multivariate analysis of overall and progression-free survival.

Parameter	Overall survival			Progression-free survival		
	p-value	Hazard Ratio	95%-CI	p-value	Hazard Ratio	95%-CI
sEGFR ≥73 ng/ml vs. <73 ng/ml	0.077	0.501	0.23–1.08	0.313	0.798	0.52–1.24
CTC counts ≥5 vs. <5 CTCs / 7.5 ml blood	**<0.001**	3.706	2.01–6.84	**<0.001**	2.349	1.55–3.56
Therapy line >1^st^ line vs. 1^st^ line	**0.001**	3.059	1.62–5.79	**<0.001**	3.188	2.11–4.82
Grading G3 vs. G1/2	**0.043**	1.334	1.01–1.76	0.161	1.149	0.95–1.40
Menopausal status Post- vs. Premenopausal	0.345	0.765	0.44–1.33	0.452	0.862	0.59–1.27
ER status Positive vs. Negative	0.136	0.547	0.25–1.21	0.058	0.584	0.33–1.02
PR status Positive vs. Negative	0.816	1.089	0.53–2.24	0.988	0.996	0.62–1.61
HER2 status Positive vs. Negative	0.110	0.624	0.35–1.11	0.430	0.855	0.58–1.26
Number of metastatic sites Multiple vs. Single site	0.970	1.013	0.51–2.02	0.338	1.266	0.78–2.05
Metastatic spread Visceral (+/−) vs. bone only	0.197	2.750	0.59–12.77	0.586	1.220	0.60–2.50
Serum HER2 Elevated vs. non-elevated	0.112	1.586	0.90–2.80	0.168	0.750	0.50–1.13

## Discussion

To our knowledge, this is the first study to address the clinical relevance of both sEGFR and CTCs in a large cohort of metastatic breast cancer patients. Previous analyses provided contradictory results on the range of normal values of sEGFR, with several studies showing decreased sEGFR levels in cancer patients compared with healthy controls, while others reported increased values in patients or found no significant differences between patients and controls^17,18,22,28^. Therefore, we performed an analysis of serum samples from 48 age-matched healthy females to determine the clinical utility of various cutoff values.

We found similar sEGFR values in healthy controls and breast cancer patients with a median value of 60.35 and 62.00 ng/ml, respectively. This is in accordance with several other studies^18,21,29^. Lafky *et al*. examined sEGFR levels using an acridinium-linked immunosorbent assay in 64 hormone receptor positive metastatic BC patients before start of a new treatment and compared them to 43 matched healthy women with no differences found between the two groups^18^. Further, Sandri *et al*. reported similar sEGFR levels in 113 metastatic BC patients and 38 healthy controls using ELISA-based assay^21^. Similar results were reported in other tumor entities, such as lung cancer^30,31^, high-grade glioma^32^ and malignant thymoma^33^. In malignant pleural mesothelioma^22^ and head and neck squamous cell carcinoma^20^ sEGFR levels were found to be significantly higher than in controls. Similarly, Tas *et al*. detected significantly higher sEGFR levels in BC patients than in healthy controls^34^. In contrast, Asgeirsson *et al*. reported significantly decreased sEGFR levels in patients with primary or metastatic breast cancer in comparison to healthy controls (primary BC: median 59 ng/ml, metastatic BC: 31 ng/ml, healthy individuals: 75 ng/ml)^1^. Decreased sEGFR levels have also been found in patients with ovarian cancer and it has been speculated that sEGFR may have potential as a screening or diagnostic test in this entity^35^.

Among the cutoff values taken into consideration, we observed the highest prognostic significance when the 75^th^ percentile, e.g. 73 ng/ml, was used. Interestingly, the cutoff previously used by other research groups, 45 ng/ml, had little clinical relevance in our patient cohort^17,23^ (Table 5). By contrast, 73 ng/ml significantly distinguished between patients with good and poor overall survival (p = 0.007 in the univariate analysis). Müller *et al*. examined serum samples from 101 patients with metastatic BC before start of chemotherapy using the same assay as in our study^17^. Patients with sEGFR levels below 45 ng/ml had a non-significant trend towards worse survival, while other cutoffs were less likely to discriminate between favorable and poor outcome. One explanation for this discrepancy might be a different disease setting: while blood samples were collected before first-line chemotherapy in all patients in the abovementioned trial, 61% of patients in our study were scheduled to begin second- or later-line of treatment. Several other trials used 45 ng/ml as cutoff and did not evaluate other values. Souder *et al*. used a similar cutoff value (44.1 ng/ml) in 535 metastatic BC patients and observed a significantly shorter overall survival in those with decreased sEGFR levels^24^. In contrast to our study, serum samples were examined before start of first-line endocrine therapy in all patients and only postmenopausal patients were enrolled in this trial. Sandri *et al*. used 45 ng/ml as cutoff because it was the lowest value in a cohort of 38 healthy controls and observed significantly worse survival in patients with decreased sEGFR^21^. However, another study using the cutoff of 45 ng/ml reported no significant impact of sEGFR levels on the clinical outcome^23^.

**Table 5:** Current evidence regarding serum EGFR in metastatic breast cancer.

Study	Setting	Patients n	Method	Follow up [months]	Cutoff	Prognostic significance
Our study	Metastatic	252	ELISA	19	73 ng/ml	Univariate: OS: yes (worse OS in patients with lower sEGFR) PFS: no Multivariate: OS: No PFS: No
Müller *et al*.^17^	Metastatic 1^st^ line	101	ELISA	8.9	45 ng/ml	Univariate: OS: trend towards worse OS in patients with lower sEGFR (p = 0.08), significant only in ER-pos patients PFS: no
Souder *et al*.^24^	Metastatic or locally advanced, ER and/or PR-pos	535	ELISA	n.a.	44.1 ng/ml	Univariate: OS: yes (worse OS in patients with lower sEGFR) Multivariate: OS: yes
Lafky *et al*.^18^	Metastatic ≥3^rd^ line	64	ALISA	n.a.	n.a. (median 3.77 fmol/ml)	Univariate: OS: No PFS: No
Sandri *et al*.^21^	Metastatic	113	ELISA	n.a.	45 ng/ml	Univariate: OS: Yes PFS: Yes
Rocca *et al*.^54^	Non-metastatic	119	ELISA	93	n.a.	Univariate: DFS: No Postoperative decrease of ≥8.65 ng/ml: worse DFS
Witzel *et al*.^23^	Metastatic	76	ELISA	18	45 ng/ml	Univariate: OS: No
Tas *et al*.^34^	Non-metastatic and metastatic	96	ELISA	19.4	n.a.	Univariate: OS: No

Although the evidence on the prognostic relevance of sEGFR is not conclusive, the majority of published studies, including the present trial, showed worse clinical outcome in BC patients with low sEGFR. This observation might seem at first surprising, since (over)expression of EGFR in the tumor tissue has been shown to predict poor survival^13–15,36^. The largest analysis of EGFR status in tumor tissue has been reported by Rimawi *et al*.^37^. 18% of 2,567 stage I-IIIA BC patients had EGFR-positive tumors; EGFR expression in patients who received adjuvant systemic therapy significantly correlated with worse DFS and OS, whereas no correlation was found in untreated patients, suggesting that EGFR expression may be associated with resistance to some forms of systemic treatment. However, one has to keep in mind that all patients whose tumors were evaluated were diagnosed between 1984 and 1999, explaining why a large proportion (46%) of patients received no systemic therapy. Nieto *et al*. evaluated tissue EGFR expression in 225 patients with locally advanced BC; patients with EGFR expression had significantly shorter relapse-free and overall survival compared to patients with no expression^14^. The multivariate analysis confirmed tissue EGFR as an independent prognostic factor. Similar results have been reported in early BC: DiGiovanna *et al*. demonstrated a negative impact of EGFR expression on the disease-free and disease-specific survival in a large cohort of 802 patients^15^. Interestingly, EGFR overexpression in this study was linked to activation of HER2, suggesting a potential role for treatment regimens targeting EGFR and HER2 in patients with co-expression of both receptors.

Few studies compared EGFR expression in tumor tissue and sEGFR. Witzel *et al*. assessed EGFR expression in 76 malignant breast tumors using immunohistochemistry and compared it to EGFR levels in serum determined using ELISA^23^. Median sEGFR levels at the diagnosis of metastatic BC did not differ between patients with EGFR expression and those with no EGFR expression in their primary tumor.

Different hypotheses have been proposed to explain why higher levels of circulating sEGFR predict longer survival, while expression of tissue EGFR is a negative prognostic factor. Obviously, sEGFR is not a simple surrogate of tumor cell load. Indeed, patients with high levels of CTCs were more likely to have decreased sEGFR in our patient cohort. Since this is the first study to measure both CTCs and sEGFR in metastatic BC, we can only speculate on the possible origins of sEGFR. Obviously, CTCs are not likely to be the main source of sEGFR detected. Since other tissues than the tumor itself may produce sEGFR, serum levels might be influenced by modified regulation through the endocrine or paracrine activity of the tumor^23^. Baron *et al*. demonstrated that gonadotropic and steroid hormones may modulate EGFR expression *in vivo*, leading to higher sEGFR levels in healthy premenopausal women than in age-matched men and postmenopausal women^38^. Further, it has been speculated that cancer cells with increased malignant potential might show a decreased proteolytic cleavage of the extracellular domain of EGFR^17^. In the context of methodology, we cannot exclude the possibility that different splice variants of sEGFR are released to the serum affecting the results of different assays used. Indeed, Baron *et al*. developed an acridinium-linked immunosorbent assay (ALISA) to measure sEGFR and reported a broader range of sEGFR concentrations than using the commercially available ELISA kits. Most importantly, the values obtained by the ALISA showed no association with those obtained using ELISA on identical serum samples^38,39^. Another possible explanation for the association between decreased sEGFR and worse survival has been discussed recently. Maramotti *et al*. hypothesized that some soluble forms of EGFR might have a physiological and protective role against cancer^40^. Indeed, circulating EGFR has been shown to inhibit proliferation and cell migration of non-small cell lung cancer cell lines *in vitro* ^41^. Interestingly, this effect could be observed only in wild type lines without EGFR mutations.

The most promising potential clinical application of EGFR detection that has been addressed in previous studies is the possibility to select patients who are likely to respond to EGFR-targeted therapy. Several molecules have been developed to block EGFR, such as cetuximab, or the EGFR tyrosine kinase domain, such as erlotinib and gefitinib. In breast cancer, the only approved therapy that targets EGFR is the oral tyrosine kinase inhibitor lapatinib. Although lapatinib targets the activity of both EGFR and HER2 receptor, only the HER2 overexpression is used to identify patients who might derive treatment benefit. Several studies evaluated the role of EGFR expression in predicting response to therapy. In a cancer cell line-based model, Zhang *et al*. found that sensitivity to lapatinib was independent of EGFR expression level in HER2-positive breast cancer cells^42^. Similarly, the EGF103009 trial confirmed that patients with HER2-positive inflammatory BC benefit from lapatinib but no clinical activity was observed in patients with EGFR-positive HER2-negative tumors, suggesting that lapatinib exhibits antitumor effects through HER2 receptor rather than EGFR. Whether sEGFR levels predict or influence the response to EGFR-targeted antibodies, remains unclear. It has been speculated that some isoforms of circulating sEGFR may serve as an alternate target for such treatment. Wilken *et al*. demonstrated that two EGFR-directed antibodies, cetuximab and panitumumab, recognize and bind sEGFR even at very low doses, suggesting that interactions between sEGFR and antibodies may be relevant in calculating the effective dose of these drugs in cancer patients^43^.

Several studies aimed at exploring the relevance of EGFR expression of CTCs. EGFR-positive CTCs has been detected in a number of tumor types, such as breast, prostate, colorectal, and lung cancer^44^. Payne *et al*. measured CTCs at different time points in a cohort of 33 metastatic BC patients and found consistent positivity over time using the CellSearch system^45^. Further, two studies examined CTC dynamics in metastatic BC patients treated with tyrosine kinase inhibitors^46,47^. Agelaki *et al*. performed CTC monitoring using immunocytochemical staining for HER2 and EGFR in patients treated with lapatinib^46^. Interestingly, the percentage of HER2-positive CTCs decreased during treatment but more patients harbored EGFR-positive CTCs at disease progression than at time of enrollment. Possibly, EGFR expression might contribute to resistance to lapatinib. These observations are further supported by a case report on a patient with progressive metastatic disease whose prolonged response to lapatinib was reflected by a striking decrease in EGFR-positive CTCs^48^. Kalykaki *et al*. reported on 17 metastatic patients with detectable CTCs after the completion of prior treatment who received maintenance therapy with gefitinib^47^. CTC counts decreased by 73% after the first cycle of gefitinib treatment. In patients initially presenting with EGFR-positive CTCs, most detected CTCs after therapy became EGFR-negative. Beyond metastatic disease, several studies addressed the relevance of EGFR status of CTCs in primary BC. Preliminary data suggest an association between EGFR positivity of CTCs and impaired clinical outcome^49^. Nadal *et al*. examined blood samples from 89 patients with localized disease and found a decrease in EGFR-positive CTCs during adjuvant chemotherapy^50^. Remarkably, patients with hormone receptor positive tumors were significantly more likely to present with EGFR-positive CTCs (33% vs. 9% in hormone receptor negative patients), supporting the biological relevance of the cross-talk between growth factor receptor- and ER-mediated pathways for the development of resistance to endocrine therapy^51^. It has been hypothesized that another pathway might be of relevance for EGFR expression as well. Kallergi *et al*. analyzed blood samples from 32 CTC-positive BC patients and showed EGFR to be co-expressed with phosphorylated EGFR, pPI3K and pAkt, implying the importance of an activated pathway in CTCs downstream of EGFR that would involve both Akt and PI3K^52^.

## Conclusions

In our study, sEGFR levels in a large cohort of metastatic breast cancer patients did not differ from those detected in healthy controls. Patients with levels above the 75^th^ percentile (73 ng/ml) had a significantly better overall survival but this association was no longer significant when CTC counts, an established biomarker in metastatic BC, were taken into account. Currently, the potentially most promising indication for EGFR measurements is the prediction of response to therapy with drugs targeting the EGFR or other erbB receptors. In the context of tailored treatment, future studies should clarify whether sEGFR levels or expression of EGFR on CTCs may identify patients likely to benefit from EGFR-targeted molecules.

## Methods

252 metastatic breast cancer patients from nine German Breast Cancer Centres were enrolled in this prospective, multicentre, open-label, non-randomized study. Blood was drawn before the start of a new line of therapy. Further inclusion criteria were: age 18 years and older, and first diagnosis of metastatic disease or disease progression before start of a new treatment line. Patients with a second primary malignancy (except *in situ* carcinoma of the cervix or adequately treated cutaneous basal cell carcinoma) were excluded. Blood samples were collected before start of a new line of therapy chosen according to national and institutional standards. Response to therapy was evaluated by computed tomography every 12 weeks. Informed consent was obtained from all individual participants included in the study.

### Quantitative analysis of serum EGFR

sEGFR was quantified by a commercially available ELISA (Oncogene Science, formerly Siemens Medical Solutions Diagnostics, now Wilex Inc., MA, USA). This sandwich-type immunoassay uses a mouse monoclonal capture antibody and an alkaline phosphatase labeled mouse monoclonal as detector. Both capture and detector reagents specifically recognize the ECD of EGFR. The capture antibody recognizes a protein domain on the extracellular portion of EGFR, does not inhibit EGF binding, and does not cross react with erbB-2 oncoprotein or human blood group A antigen. To perform the test, an appropriate volume of specimen is incubated in the wells to allow binding of the antigen by the capture antibody. The immobilized antigen is then exposed to the alkaline phosphatase labeled detector antibody. Addition of substrate to the wells allows the catalysis of a chromogen into a colored product, the intensity of which is proportional to the amount of EGFR which has been bound to the plate. Using a microtiter plate reader, the absorbance of the colored product in the Standards and sample wells can be measured simultaneously. Correlating the absorbance values of samples with the Standards allows the investigator to determine the levels of EGFR in a sample. Samples may be assigned a quantitative value of sEGFR in nanograms per mL (ng/ml) of serum or plasma. For the determination of the cutoff, blood samples from 48 age-matched healthy controls were analyzed (Table 2). The sEGFR concentration was estimated from the standard curve. Each sample, standard and control were analyzed in duplicate.

### Detection of other biomarkers

CTCs were detected using the CellSearch™ system (Veridex LLC, NJ, USA). Briefly, 7.5 ml peripheral blood were collected into CellSave Tubes and processed according to manufacturer’s instructions. The assay consists of an immunomagnetic enrichment step employing immunomagnetic beads coated with anti-epithelial cell adhesion molecule (EpCAM) antibody, followed by staining with several antibodies. A circulating tumor cell is defined as a CD45-negative cytokeratin-positive cell with a DAPI-stained nucleus. In the current study, CTC-positive patients were defined as those with at least five tumor cells per 7.5 ml blood. Serum HER2 was determined using a commercially available ELISA (Martell Diagnostic Laboratories, Roseville, MN, USA; formerly Wilex Inc, Cambridge, MA, USA), as described previously^16^. This test is based on the quantitative measurement of the ECD of the HER2 protein and uses one mouse monoclonal antibody to capture the extracellular domain and another one to detect and quantify it. The assay has been cleared by the Food & Drug Administration (FDA) with the recommended cut-off of 15 ng/ml.

### Statistical analysis

Chi-squared test and Fisher’s exact test were used to evaluate the relationship between EGFR detection and clinical-pathological factors. The means between the control group and patients were compared using independent samples t-test. In the survival analysis, following primary end points were considered: (1) death and (2) progression. Survival intervals were measured from the time of blood sampling to the time of death or of the first clinical, histological or radiographic diagnosis of progression. We constructed Kaplan–Meier curves and used the log-rank test to assess the univariate significance of the parameters. Cox regression analysis was used for multivariate analysis. All reported p-values are two-sided. Statistical analysis was performed by SPSS, version 18 (SPSS Inc., Chicago, IL, USA). The analysis was performed according to the REporting recommendations for tumor MARKer prognostic studies (REMARK) criteria on reporting of biomarkers^53^. The primary question was the prognostic impact of sEGFR in the entire patient cohort.

### Data availability statement

The datasets generated during the current study are available from the corresponding author on reasonable request.

### Ethical approval

All procedures performed in this study were in accordance with the ethical standard of the institutional and national research committee and with the 1964 Helsinki declaration and its later amendments or comparable ethical standards. The study was approved by the local ethical committees of participation institutions.

### Informed consent

Informed consent was obtained from all individual participants included in this study.
